# Dermal Microvascular Units in Domestic Pigs (*Sus scrofa domestica*): Role as Transdermal Passive Immune Channels

**DOI:** 10.3389/fvets.2022.891286

**Published:** 2022-04-25

**Authors:** Xiangfei Meng, Zhaoxuan Zhu, Nisar Ahmed, Qianhui Ma, Qi Wang, Bihua Deng, Qiusheng Chen, Yu Lu, Ping Yang

**Affiliations:** ^1^MOE Joint International Research Laboratory of Animal Health and Food Safety, College of Veterinary Medicine, Nanjing Agricultural University, Nanjing, China; ^2^Department of Veterinary Anatomy and Histology, Faculty of Veterinary and Animal Sciences, Lasbela University of Agriculture, Water & Marine Sciences (LUAWMS), Uthal, Pakistan; ^3^National Research Center of Engineering and Technology for Veterinary Biologicals, Institute of Veterinary Immunology and Engineering, Jiangsu Academy of Agricultural Sciences, Nanjing, China

**Keywords:** dermal microvascular unit, domestic pigs (*Sus scrofa domestica*), macrophages, jet needle-free injection, skin immunity, veil cells, telocyte

## Abstract

The dermal microvascular unit (DMU) is a perivascular functional unit in the dermis. It is composed of microvascular and capillary lymphatics surrounded by immune cells. In this study, jet needle-free injection system was used to injected biocompatible carbon nanoparticles into the cervical skin of domestic pigs (*Sus scrofa domestica*) and assessed the morphological distribution of DMUs by hematoxylin erythrosine staining, immunohistochemistry (IHC), and transmission electron microscopy (TEM), and TEM was also used to observe the ultrastructural changes of DMUs after jet needle-free injection. Following our study, we identified DMUs in the dermis stratum papillare and similar structures in the dermis stratum reticulare, but the aggregation of CD68^+^ and CD1a^+^ cells in the dermis stratum papillare of DMUs by IHC confirmed that DMUs act as reservoirs of dermal immune cells, while similar structures in the dermis stratum reticulare should not be considered as DMUs. Ultrastructure of DMUs was revealed by TEM. Marvelous changes were found following xenobiotics attack, including the rearrangement of endothelial cells and pericytes, and the reactivity of immune cells. Novel interstitial cell telocyte (TC) was also identified around the microvasculature, which may have been previously known as the veil cell. Our results successfully identified the distribution of DMUs in the skin of domestic pigs, which might act as reservoirs of immune cells in the skin and play a role in immune surveillance and immune defense.

## Introduction

The skin is the first line of defense against the outside world in higher vertebrates. Not until Streilein introduced the concept of skin-associated lymphoid tissue (SALT) did people regard the skin as having dual roles as physical barrier and immune function. It is found that the dermis is the major site where immune function occurs and contains twice as many immune cells in the circulatory system. These immune cells are not randomly distributed in the dermal structures, but share a close anatomical relationship with the microvessels of the dermal superficial vascular plexuses (DSVPs) (1, 2). The dermal microvascular unit (DMU) is the basic model for this constellation of dermal cells centered on the vasculature. In the original study, DMUs were considered to contain dermal microvascular endothelial cells (DMECs), dermal perivascular dendrocytes (DPDCs, including dendritic-like macrophages and dendritic cells), dermal perivascular T cells (DPTCs), dermal perivascular mast cells (DPMCs) and other cells unrelated to immunity (e.g., fibroblasts, and pericytes), which could not completely reveal the mechanism of its involvement in cutaneous immunity as a functional unit. Therefore, recent studies have complemented the original theory by including capillary lymphatics, which share an intimate anatomical relationship with these structures, in DMUs (3, 4).

Since capillary lymphatics and capillaries are not easily identifiable in routine histological evaluation, electron microscopy remains the best method for identifying DMUs (5). Ultrastructural evidences suggest that the center of DMUs is composed of DMECs that originate from the horizontal papillary plexuses (6). These structures are responsible for the blood supply to the dermis stratum papillare and are responsive to injury (7), hypoxia (8), and stress (9, 10), which manifests in the ultrastructure by gap formation and altered deposition in the basement membrane material of the vascular wall (11–13). Pericytes are located adjacent to or above endothelial cell junctions, which can control the contraction of DMECs by upregulating endothelin-1 (ET-1) and downregulating of iNOS expressed by DMECs (14, 15). During inflammation, pericytes can cover the endothelial cell gap through rearrangements, which is important in the study of skin pathology (16, 17). The existence of DPDCs, DPTCs and DPMCs located on the periphery of pericytes allows the DMU to act as a functional unit of cutaneous immunity, these cells accumulate near DMECs and serve as a possible reservoir for lymphocyte recirculation in the skin (18, 19). The capillary lymphatics that comprise belonging to DMUs are superficial dermal lymphatic plexuses and not mentioned in the manuscript originally introducing the concept of DMUs, but their unique immunological role in the dermis has encouraged the possibility of exploring them as part of DMUs (3, 4). It has been found that during the inflammatory or immune response phase, lymphendothelial cells (LECs) guide the directional migration of dermal dendritic cells (d DCs, 10–15%) and T cells (80–90%) *via* CCL21 and sphingosine-1 phosphate (S1P) signaling, and LYVE-1 has also been identified to guide the migration of d DCs (20–23).

Domestic pigs (*Sus scrofa domestica*) are economically important animals and a major source of meat in many countries. Vaccination of domestic pigs is usually performed by injection, but this may cause lesions in pork carcasses and losses to the pig industry (24). In recent years, transdermal immunization methods including transdermal delivery system (TDS), transdermal needle-free injection (NFI) and solid microstructured transdermal system (sMTS) have endeavored to solve the problems caused by traditional injections, which have been used on a small scale with good effects (25–27). However, there are no reports confirming the presence of DMUs in domestic pigs, and even a few studies have dug deep into the ultrastructural changes of DMUs during xenobiotics (i.e., antigenic or non-antigenic substance) attack on the skin of domestic pigs. In this study, the presence of DMUs in domestic pig skin was confirmed for the first time by histological analysis and immunohistochemistry (IHC), and DMUs were identified as reservoirs of immune cells for the first time. In addition, transmission electron microscopy (TEM) was employed to analyze the ultrastructural changes of DMUs after attack of the skin by xenobiotics, which have elucidated the ultrastructure of DMUs and validated the potential role of DMUs involved in passive skin immunity. Our results will contribute to a profound understanding of the mechanisms of passive skin immunity under foreign substance attack and provide new ideas for the development of transdermal immunization methods in domestic pigs.

## Materials and Methods

### Animals and Ethics Statement

Three-month-old male domestic pigs, 45 to 49 kg, were purchased from Jiangsu Zhongcheng Company (Yancheng, China). Pigs were randomly divided into two groups: Control and 5% biocompatible carbon nanoparticles, five pigs in each group. Under pentobarbital sodium (30 mg/kg, Sinopharm Chemical Reagent Co., Shanghai, China) anesthesia, biocompatible carbon nanoparticles was injected into the cervical skin by group through the POK-MBX jet needle-free injection system (DERM-G1-2, German Derm Co., Hong Kong, China), and the control group was left untreated. One hour later, animals were sacrificed and the cervical skin was collected.

The study protocol was approved by the Animal Ethics Committee of Nanjing Agricultural University. The animals were housed in the experimental animal center of Nanjing Agricultural University and leave libitum access to filtered water and food, adaptive feeding was given for 5 days before the experiment.

### Histological Evaluation

Cervical skin was fixed in 4% paraformaldehyde for 48 h, embedded in paraffin, sectioned at a thickness of 7 μm. After deparaffinization, the sections were stained with hematoxylin eosin staining solution (Leagene biotechnology, Beijing, China) for light microscopic analysis using Olympus microscope (DP73, Tokyo, Japan).

### Immunohistochemistry

Paraffin sections (7 μm) were deparaffinized on positively charge slides, blocked endogenous peroxidase with 3% hydrogen peroxide for 10 min at 37 °C, and used sodium citrate buffer (0.01 M, pH 6.0, 95°C) to expose antigenic epitopes. Treated samples were blocked with 5% bovine serum albumin for 40 min (BSA, Boster Biological Technology, Wuhan, China) and incubated with rabbit anti-CD68 antibody (1:100, Boster Biological Technology, Wuhan, China), rabbit anti-CD1c antibody (1:100, Bioss Biological Technology, Beijing, China), and rabbit anti-CD1a antibody (1:100, Boster Biological Technology, Wuhan, China) overnight at 4°C, negative controls were set up with phosphate buffered saline (PBS, 0.1 M, pH 7.4) instead of primary antibody. The next day, samples washed with PBS (0.1 M, pH 7.4) were incubated with anti-rabbit IgG antibody (1:100, Servicebio Technology, Wuhan, China) at 37°C for 1 h, washed again and analyzed for peroxidase activity with diaminobenzidine (DAB, Boster Biological Technology, Wuhan, China), and nuclei were stained with hematoxylin.

### Transmission Electron Microscopy

Skin tissue sectioned to 1 mm^3^ in size was fixed overnight in 2.5% glutaraldehyde at 4°C. After PBS (0.1 M, pH 7.4) rinsing, 1% osmium tetroxide (Polysciences Inc. Warrington, Pennsylvania, USA) was used to fix the tissue at room temperature. Following the dehydration treatment, the tissue was embedded in Epon812 (Merck & Co Inc., New Jersey, USA) for 3 days at 60°C. The treated tissue samples were fine-sliced into 50 nm ultrathin sections and anchored on copper grids. Sections were stained with uranyl acetate and lead citrate, observed under the Hitachi TEM system (H-7650, Tokyo, Japan).

### Statistical Analysis

Ten randomly selected DMUs and similar structure consisting of deep dermal vascular plexuses (DDVPs) in the dermis stratum reticulare from the same immunohistochemical section were photographed under the same field of view, and gray-scale analysis of the integral optical density (IOD) was done by using Imagepro Plus 6.0. Statistical differences between the two groups were analyzed by student's *t*-test (R version 4.1.1 and Rstudio version 1.4). The ggplot 2 visualization package (version 3.3.5) for the R (version 4.1.1) programming language was used to generate box plots, *P* < 0.05 was considered statistically significant.

## Results

### Distribution and Identification of DMUs

**Figure 1:**
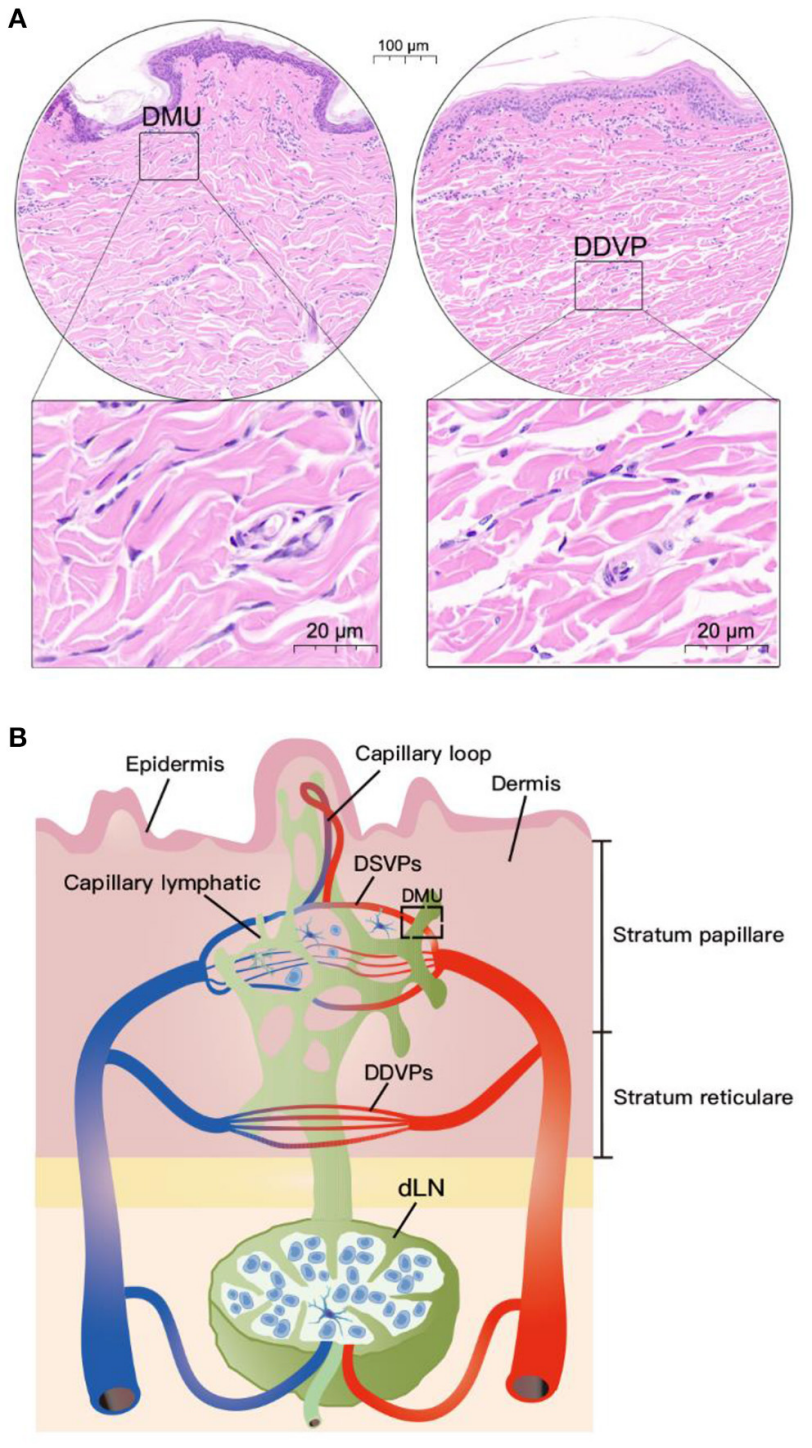
Location of DMUs in the skin with HE staining. **(A)** Localization of DMUs as well as DDVPs in the skin; **(B)** Describe the distribution and structure of DMUs in the skin. DMU, dermal microvascular unit; DSVPs, dermal superficial vascular pleauxes; DDVPs, deep dermal vascular plexuses; dLN, draining lymph node; Scale bar in circular region denotes 100 μm and scale bar in square area denotes 20 μm.

HE results of pig neck skin showed that the DMU was composed of capillary lymphatic, microvessel and peritubular cells (Figure 1A). However, the morphology of capillary lymphatics in HE results is often “collapsed” and difficult to identify. Nevertheless, we can observe that DMUs were observed to be widely distributed in the dermis, mainly in the papillary dermis near the epidermis (Figure 1A) rather than in the dermis stratum reticulare. Although blood vessels and lymphatics are also distributed in the dermis stratum reticulare, they tend to be separate and do not function in concert (Figure 1A). Based on previous knowledge and this study, we drew a model diagram to describe the spatial position of the DMUs in the microcirculation: Blood vessels and lymphatic vessels are widely distributed in the dermis, with the DMUs found mainly in the papillary dermis, consisting of a network of microvessels belonging to DSVPs and lymphatic vessels belonging to the superficial dermal lymphatic plexuses. The reticular dermis is blood supplied by DDVPs and few immune cells existed, although it also has a similar structure of intertwined vascular lymphatic vessels. Immune cells in the skin can enter the draining lymph nodes through DMUs from lymphatic vessels or high endothelial microvessels (Figure 1B).

### Immunological Structural Characteristics of DMUs

**Figure 2:**
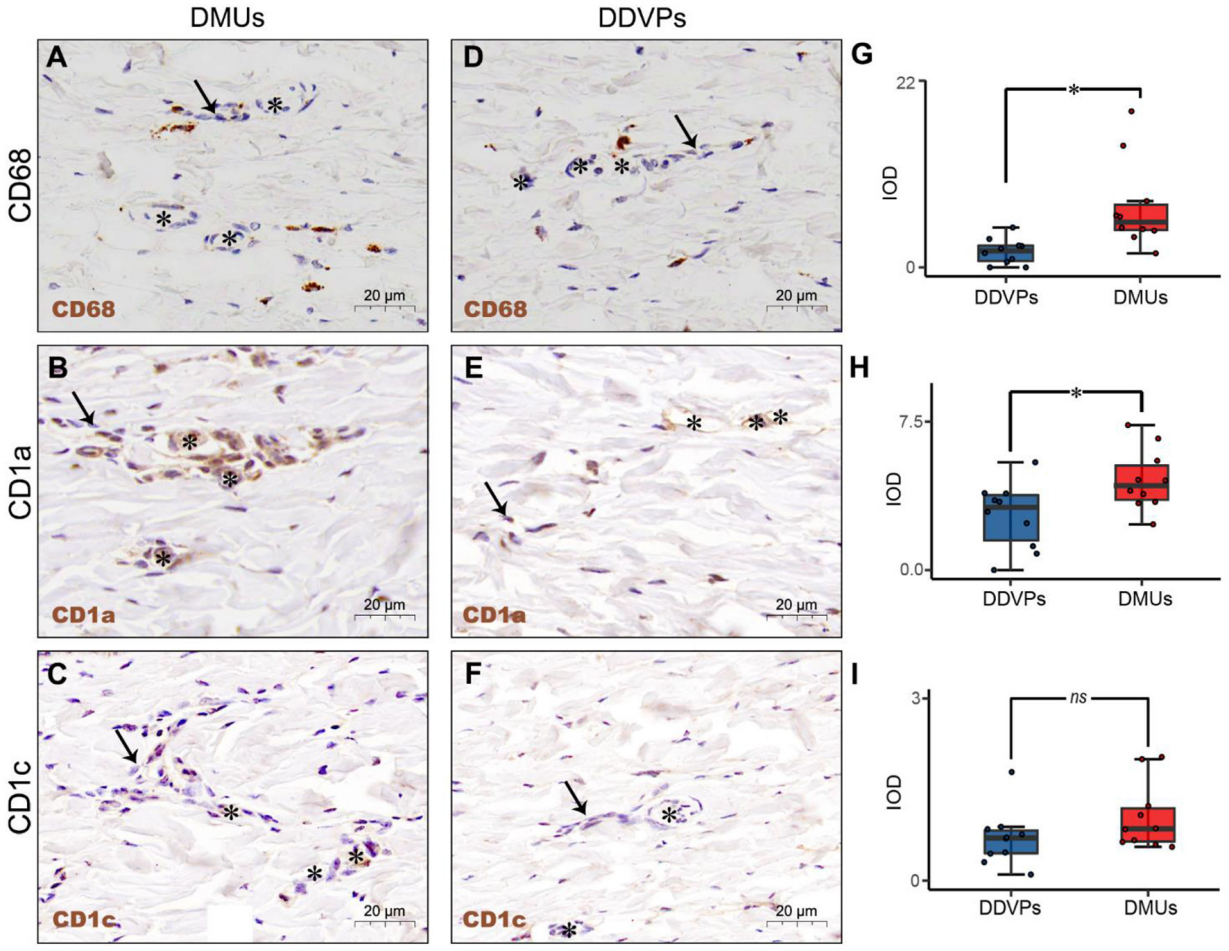
The immunological structure of DMUs identified by IHC. **(A)** DMUs in pig cervical skin marked by CD68; **(B)** DMUs in pig cervical skin marked by CD1a; **(C)** DMUs in pig cervical skin marked by CD1c; **(D)** DDVPs in pig cervical skin marked by CD68; **(E)** DDVPs in pig cervical skin marked by CD1a; **(F)** DDVPs in pig cervical skin marked by CD1c; **(G)** CD68^+^ expression quantified by IOD in DMUs and DDVPs; **(H)** CD1a^+^ expression quantified by IOD in DMUs and DDVPs; **(I)** CD1c^+^ expression quantified by IOD in DMUs and DDVPs. **(J)** The anatomical location of DMUs in the skin and the structure of DMUs are presented in a schematic diagram. *ns* indicates no significant difference; *P < 0.05 indicates significant difference. Asterisks (*) point to microvasculature and arrows (→ ) point to capillary lymphatics; DMU, dermal microvascular unit; DDVPs, deep dermal vascular plexus; IOD, integrity optical density; scale bar **(A–E)** = 20 μm.

IHC was used to identify the relationship between immune cells and DMUs in the dermis. We labeled immune-associated cells in DMUs with CD68, CD1a, and CD1c. CD68^+^, CD1a ^+^ and CD1c^+^ cells were observed in the interwoven lymphatic and microvascular areas (Figures 2A–C). Although a small number of CD68^+^, CD1a^+^ and CD1c^+^ cells accumulated near vessels in DDVPs, they were not common and some of these immune-positive cells were not even found near DDVPs (Figures 2D–F). We performed student's *t*-test on mean IOD of the positive results presented by the IHC to show that there were significantly more CD68^+^ cells (*P* < 0.05, Figure 2G) and CD1a^+^ cells (*P* < 0.05, Figure 2H) clustered near lymphatic vessels and blood vessels of DMUs than DDVPs. CD1c^+^ cells located in DMUs were slightly more than those near DDVPs, but the statistical significance was not recognized (*ns*, Figure 2I).

### Ultrastructural Analysis of DMUs

**Figure 3:**
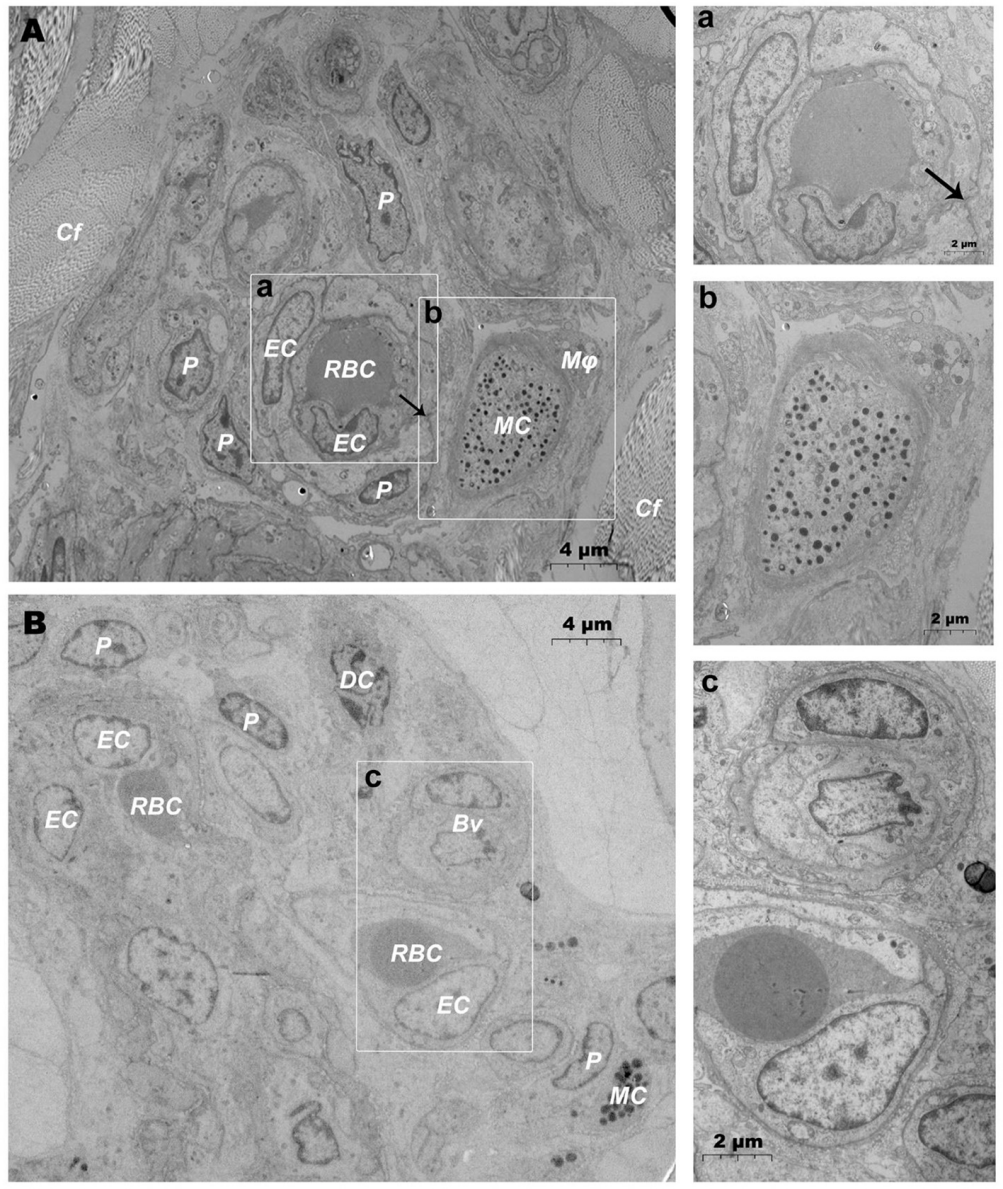
Ultrastructural analysis of microvessels at the steady state *via* TEM. **(A,B)** Ultrastructure of microvessels and surrounding cells in steady state; (a) is the magnification of the microvessel in **(A)**; (b) is the magnification of the mast cells in **(A)**; (c) is the magnification of microvessels in **(B)**. RBC, red blood cell; EC, endothelial cell; P, pericyte; MC, mast cell; Mϕ, macrophage; Cf, collagen fiber; Bv, blood vessel; DC, dendritic cell; →, Tight junctions between the pericyte and the endothelial cell. Scale bar **(A,B)** = 4 μm; (a–c) = 2 μm.

**Figure 4:**
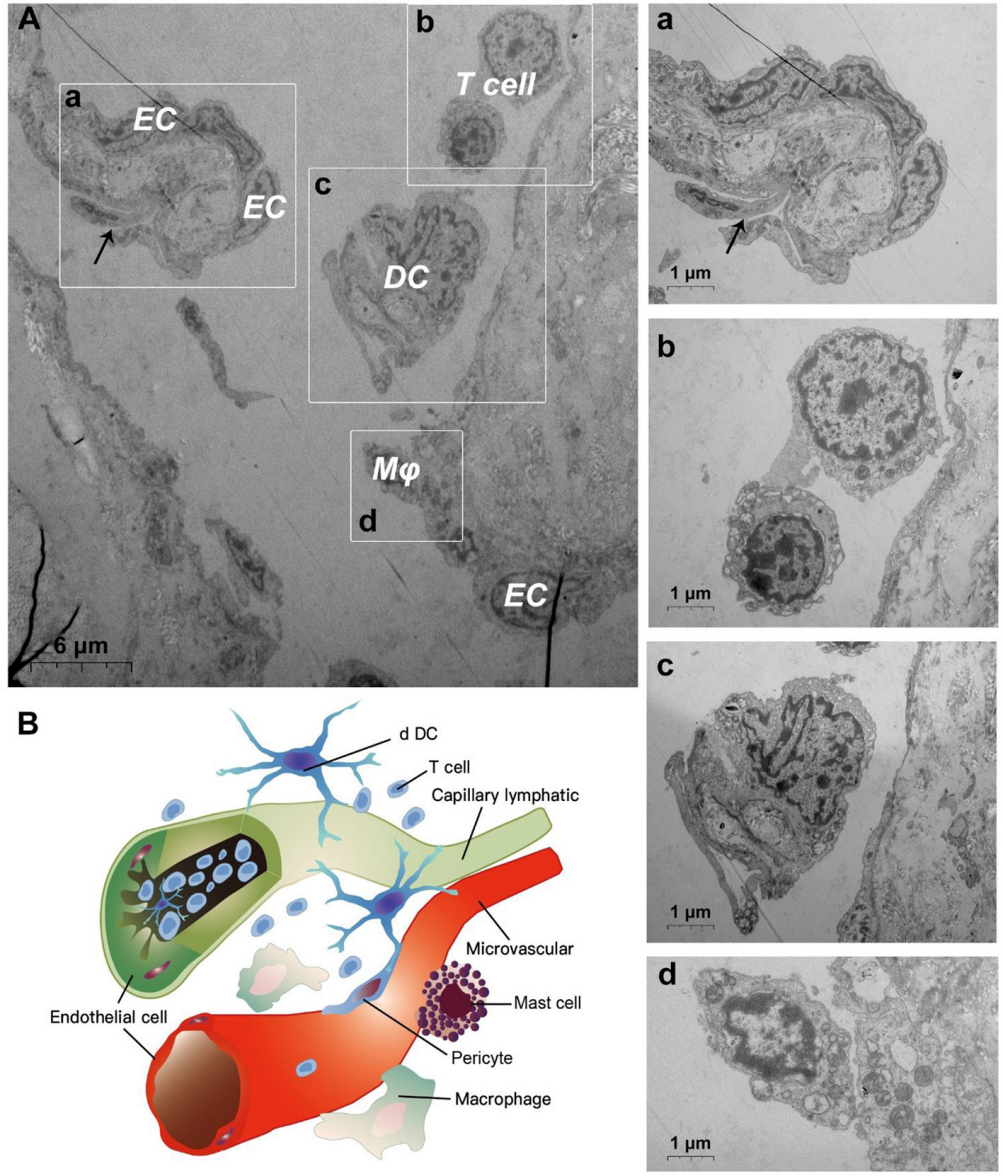
Ultrastructural analysis of lymphatic vessels at the steady state *via* TEM. **(A)** The ultrastructure of lymphatic vessel and surrounding cells in the steady state; (a) is the enlargement of lymphatic endothelium in **(A)**; (b) is the enlargement of T cells in **(A)**; (c) is the enlargement of the dendritic cell in **(A)**; (d) is the enlargement of the macrophage in **(A)**; **(B)** Pattern diagram of DMUs in the steady state. EC, endothelial cell; Mϕ, macrophage; DC, dendritic cell; →, Imbricate overlap of endothelial cells. The scale bar **(A)** = 6 μm; (a–d) = 1 μm.

TEM evaluation observed that the capillary wall was mainly composed of a layer of endothelial cells and basal membrane (Figures 3A,B). The thin cross section of capillaries was surrounded by a single endothelial cell (Figure 3c), and the thicker capillaries were surrounded by multiple endothelial cells (Figure 3a). A little connective tissue was found outside the basal membrane of endothelial cells. Kinds of flat and protuberant pericytes, which forms a tight connection with the endothelial cells through the rupture of the basal membrane were found between the endothelial cells and the basal membrane (Figure 3a). DPMCs, macrophages (Figure 3A), and d DCs (Figure 3B) were seen around the microvasculars. Among them, mast cells are oval with irregular membrane and a large number of basophilic particles can be seen in cytoplasm (Figure 3b). Capillary lymphatics were thin-walled lumens made up of monolayer lymphatic endothelial cells (Figure 4A), which have very thin cytoplasm except for the perinuclear region. The connections between LECs were loose, lacking tight connections and adhesion connections, and intercellular stacked tile-like structures at the junctions existed at adjacent endothelial cell connections (Figure 4a). The lymphatic vessels also contain a variety of cells: T cells (Figure 4b), dendritic cells (Figure 4c), and macrophages (Figure 4d). This led us to draw a pattern of DMUs: microvascular and capillary lymphatic in the region share an intimate anatomical relationship, and DPMCs, macrophages, d DCs, and T cells were present around or in the lumen of the vessels (Figure 4B).

### Ultrastructural Changes in DMUs After Jet Needle-Free Injection

**Figure 5:**
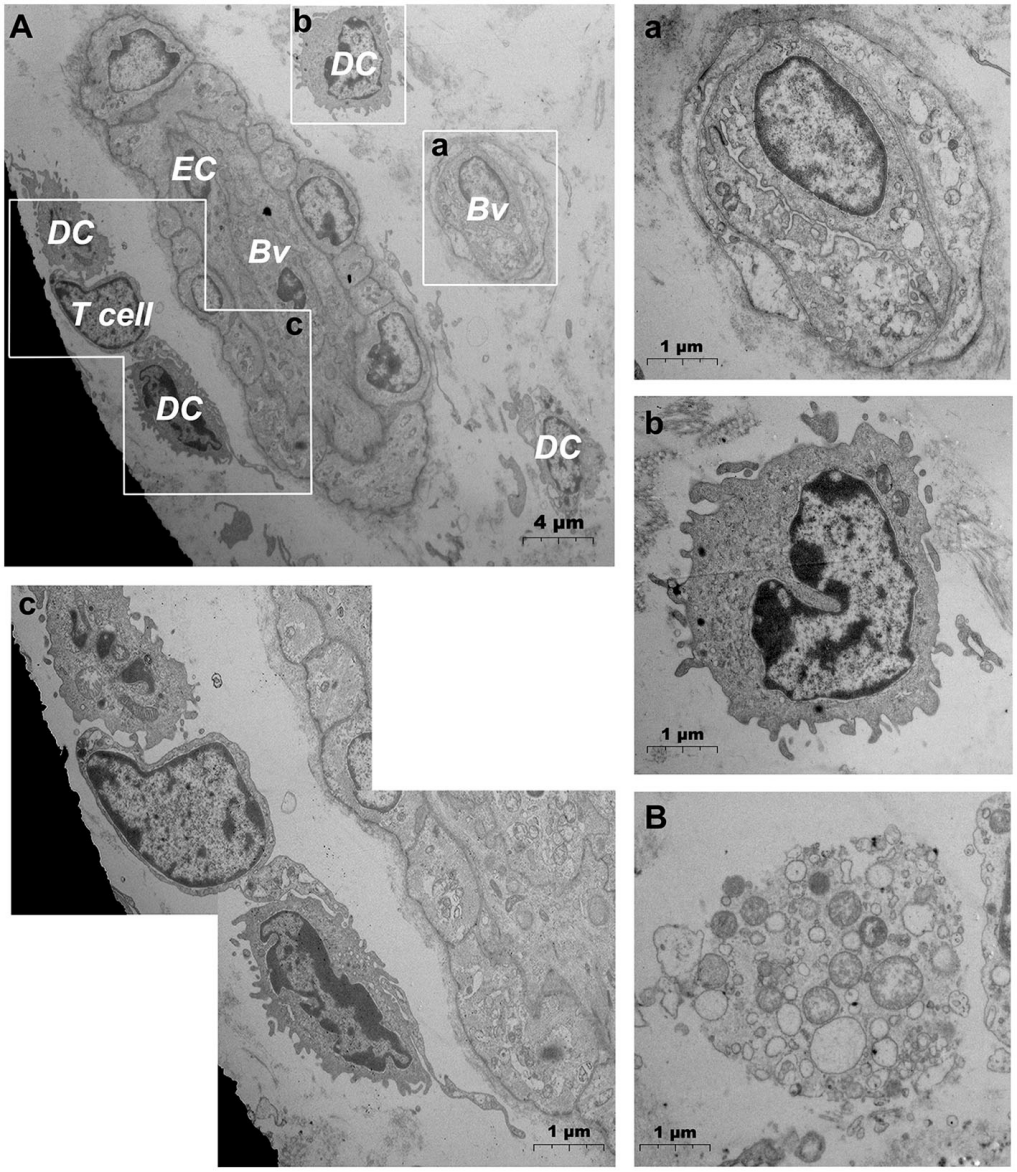
Ultrastructural analysis of microvascular groups via TEM after jet needle-free injection. **(A)** Ultrastructure of microvessels and surrounding cells after jet needle-free injection; (a) is the enlargement of the capillarie in **(A)**; (b) is the enlargement of the dendritic cell in **(A)**; (c) is the enlargement of dendritic cells and the T cell in **(A)**; **(B)** Macrophages that engulf carbon nanoparticles. Bv, blood vessel; EC, endothelial cell; DC, dendritic cell. The scale bar **(A)** = 4 μm; **(B)** (a–c) = 1 μm.

**Figure 6:**
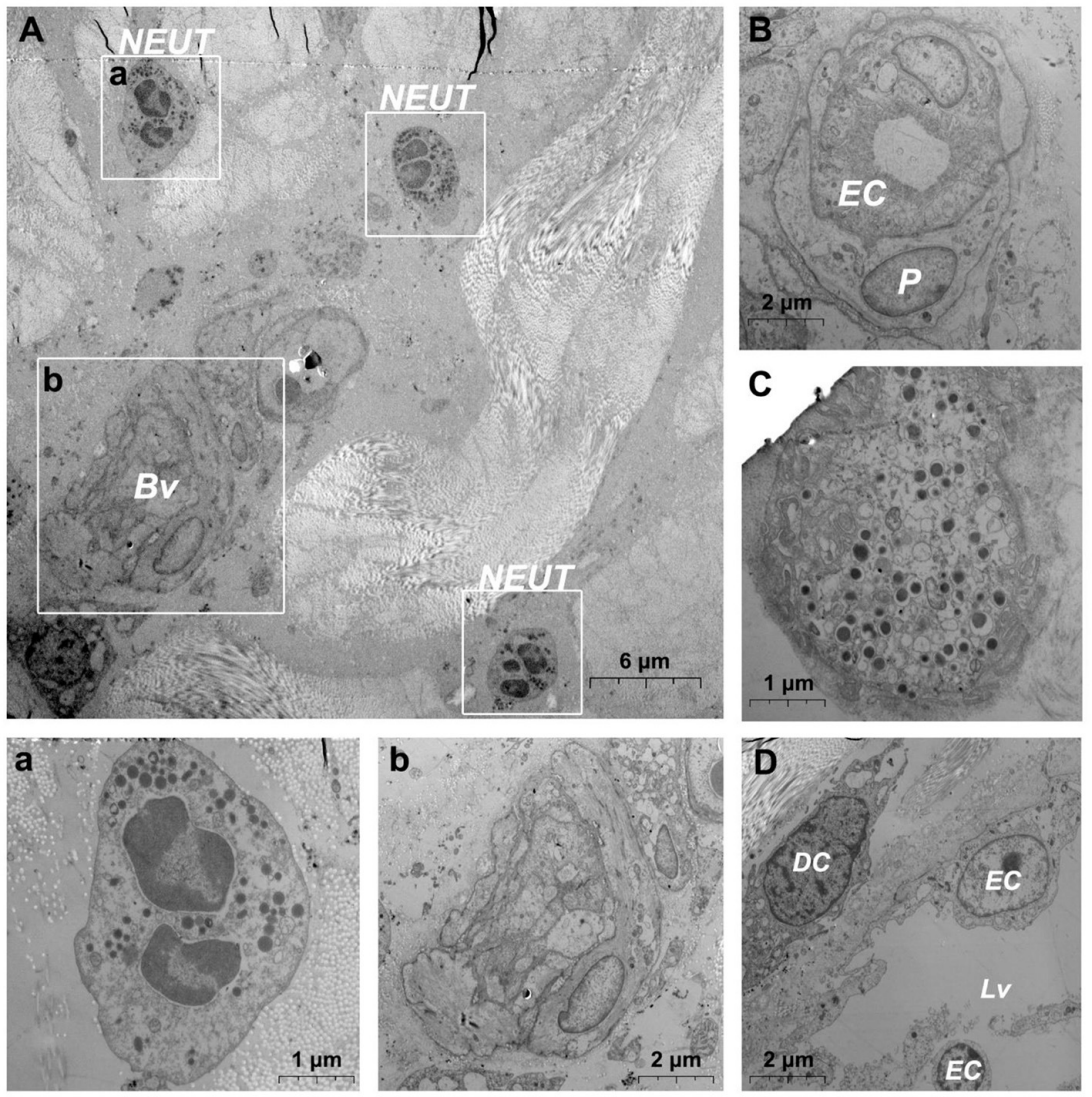
Ultrastructural analysis of the microvessel *via* TEM after jet needle-free injection. **(A)** Ultrastructure of the microvessel and surrounding cells after jet needle-free injection; (a) is the enlargement of neutrophils in **(A)**; (b) is the enlargement of blood vessels in **(A)**; **(B)** Swollen microvessels after jet needle-free injection; **(C)** Mast cell that is degranulating after jet needle-free injection. **(D)** Swollen lymphatic vessel and surrounding dendritic cell after jet needle-free injection. Bv, blood vessel; NEUT, neutrophil; DC, dendritic cell; Lv, lymphatic vessel. The scale bar **(A)** = 6 μm; **(B,D)** (b) = 2 μm; **(C)** (a) = 1 μm.

After needlefree injection of biocompatible carbon nanoparticles, we observed *via* TEM that the DMECs showed varying degrees of swelling, with increased cytoplasm in the area surrounding the nucleus (Figures 5A,a). d DCs (Figures 5b,c), T cells (Figure 5c) and macrophages (Figure 5B) surrounded the microvessels, and the number of cells gathered was larger than before. Carbon nanoparticles also appears in the phagocytes of surrounding macrophages (Figure 5B). We also observed thickening of the basement membrane of blood vessels was observed (Figure 6b), and the connection junction between DMECs and pericytes was tighter (Figure 6B). In addition, the number of perivascular neutrophils increased (Figure 6a), and magnification viewing revealed the presence of obvious nanocarbon particles around the neutrophils (Figure 6a). In addition, some of DMECs already showed degranulation (Figure 6C). Similarly, the LECs appeared significantly swollen. There were fewer cells in the lumen of the lymphatic vessels than before, but dendritic cells appeared outside the lumen (Figure 6D).

### Association Between TCs and DMUs

**Figure 7:**
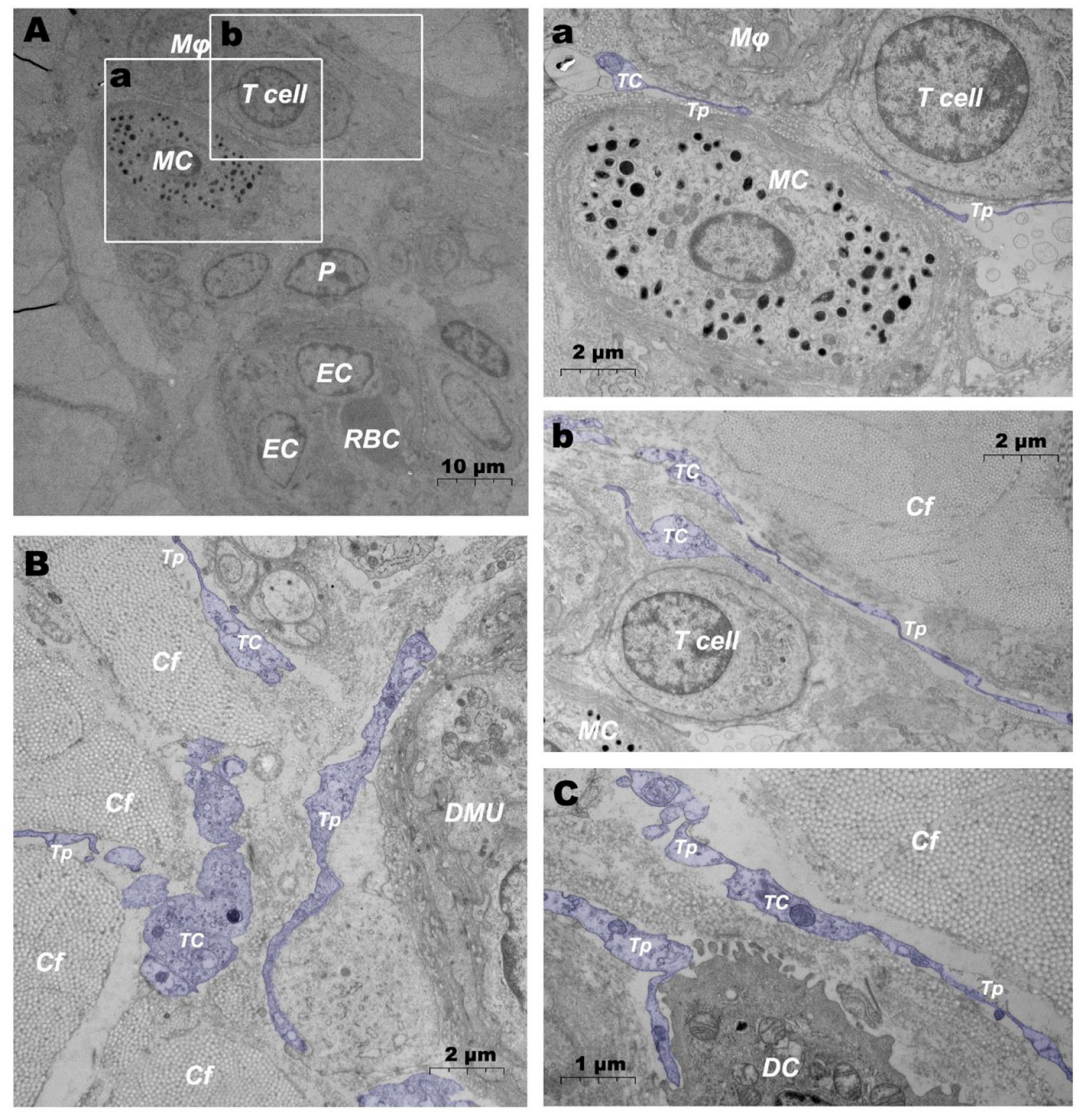
Ultrastructural analysis of TCs in the steady state *via* TEM. **(A)** Location of TC in the DMUs in the steady state; (a) is the enlargement of the mast cell and surrounding TCs in **(A)**; (b) is the enlargement of T cells and surrounding TCs in **(A)**; **(B)** TCs surrounds the DMUs; **(C)** TC between collagen fibers and dendritic cells. RBC, red blood cell; EC, endothelial cell; P, Pericyte; MC, mast cell; Mϕ, macrophage; TC, telocyte; Tp, telopode; Cf, collagen fiber; DMU, dermal microvascular unit; DC, dendritic cell; Blue mark, telocytes. The scale bar **(A)** = 10 μm; **(B)** (a,b) = 2 μm; **(C)** = 1 μm.

**Figure 8:**
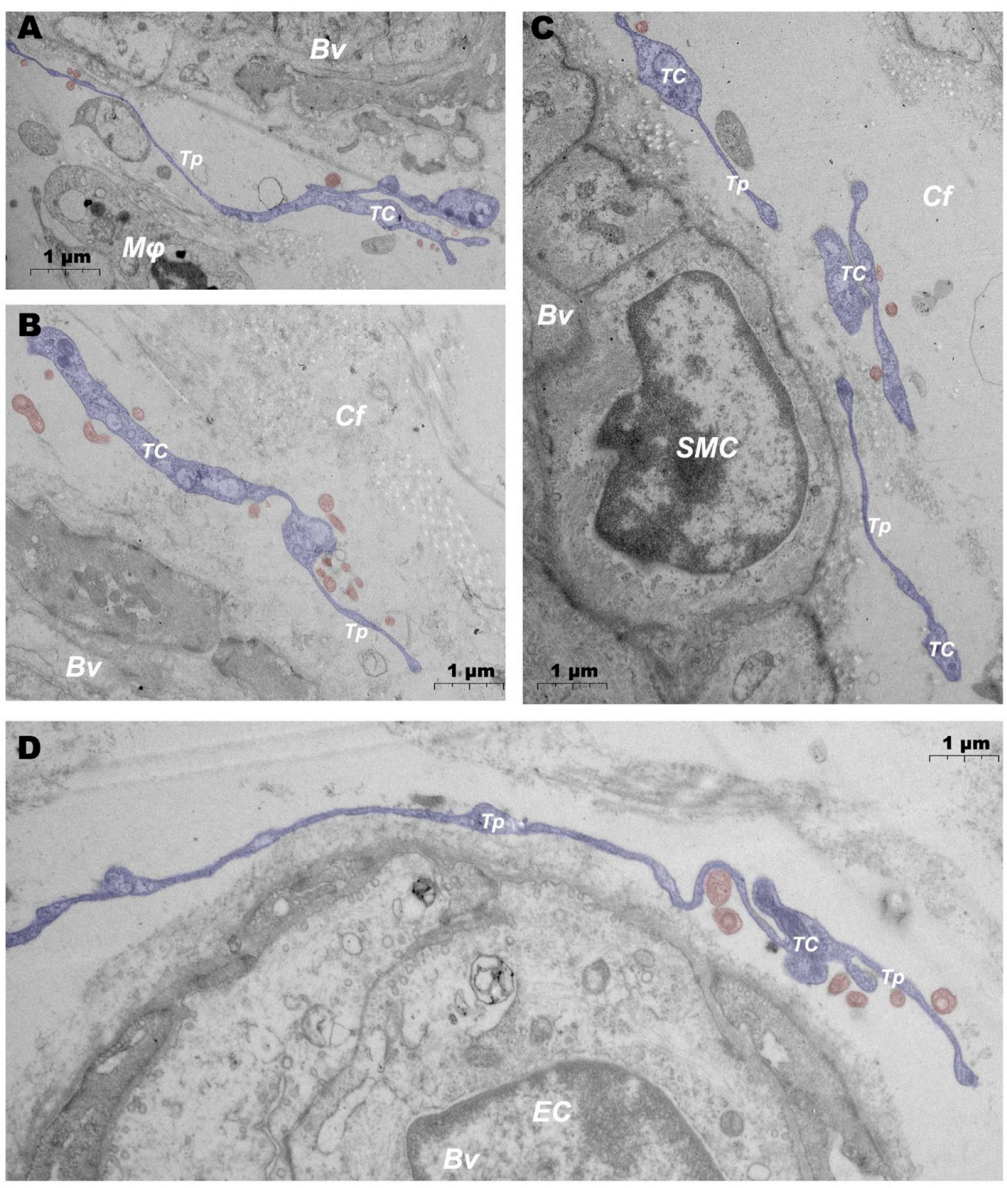
Ultrastructural analysis of TCs after jet needle-free injection via TEM. **(A–D)** After jet needle-free injection, TCs at different locations in the DMUs releases a large number of vesicles. BV, blood vessels; Mϕ, macrophage; TC, telocyte; Tp, telopode; Cf, collagen fiber; SMC, smooth muscle cell; EC, endothelial cell; Blue mark, telocytes; Red mark, vesicles. Scale bar **(A–D)** = 1 μm.

In addition, when observing DMUs, we found that interstitial cell telocyte (TC) newly discovered in recent years was inextricately related to DMUs (Figures 7, 8). The morphology and distribution of TCs are very similar to the characteristics of veil cells described in the previous article. The cell bodys of TCs are small and fusiform, with a spindle or oval nucleus whose processes extend tens or even hundreds of microns in length (Figures 7A,B). The elongated protuberations of TCs allow it to contact other components such as microvessels (Figure 7B), DPMCs (Figure 7a), T cells (Figure 7b), and d DCs (Figure 7c) in the DMUs, forming homomorphic or heteromorphic connections and complex three dimensions (3D) networks. In addition, a portion of TCs were located between DMUs and collagen fiber (Figure 7C), which may be the barrier between DMUs and the dermis. A large number of unreleased vesicles were observed in the telopodes (Tps, cytoplasmic protrusion of TCs), (Figures 7B,C). However, after jet needle-free injection of carbon nanoparticles, it was observed that the TCs around the DMUs released many vesicles of different sizes (Figures 8A–D).

## Discussion

The immune mechanism of the skin remains poorly understood despite SALT has been proposed for a long time. The dermal microvascular unit, or the DMU, a unique immune structure in the skin, is considered to be significant for the immune function of the skin (3, 4). With the innovation of modern veterinary medicine, there is a recognized need for non-invasive and efficient injection methods that can be used to reduce injection costs and prevent animal stress, and DMUs naturally became a widely acknowledged research point. An initial aim of this project was to identify the predictive ability of DMUs in domestic pigs, which is an important consideration, especially when manipulating them for immunoprophylaxis.

This is the first study that confirms that DMUs is significantly associated with intradermal immune responses based on jet needle-free injection technology in domestic pigs. These findings correlate with previous description of the ultrastructure of DMUs and further support a role of DMUs in Transdermal passive immunity. DMUs are composed of microvessels, capillary lymphatics and peritubular cells, which jointly constitute the immune units within the dermis and acts as reservoirs for skin immune cells. We also found that DMUs undergo ultrastructural changes, including endothelial cell changes and migration of immune cells, in response to xenobiotics attack. Surprisingly, a type of novel interstitial cell TC has been found to participate in the composition of DMUs, perhaps what used to be known as veil cells.

In histological analysis, DSVPs and DDVPs were identified as responsible for the blood supply to the papillary and dermis stratum reticulare, respectively, and DSVPs also have an intimate anatomical relationship with the superficial dermal lymphatic plexuses (28). CD1a, CD1c and CD68 are all faithful markers of immune cells within the skin. Interestingly, there are significantly more CD1a^+^, CD1c^+^, and CD68^+^ cells around DSVPs intertwined with superficial dermal lymphatic plexuses (i.e., DMUs) than in similar structures composed of DDVPs (28, 29). This perivascular immune structure is often mediated by vascular endothelial growthfactor receptors (VEGFR) family, monocyte chemoattractant protein-1 (MCP-1) and endogenous nitric oxide (NO) and is important evidence that DMUs play an immune surveillance role at steady state (30–33).

At steady state, immune cells in the skin of domestic pigs were found to be clustered around capillaries and in capillary lymphatics *via* TEM. Macrophages, DPMCs, d DCs and T cells were regularly distributed around the microvasculature. DPMCs were often found at the peripheral edge of the pericytes, and intercellular gap junctions were observed. In contrast, other immune cells were often located at the periphery of mast cells, within collagen fibers. We also noted immune cells within the stacked LECs, which lack the regularity of the vascular microenvironment but bridging the skin to the draining lymph nodes. Recent studies have indicated that DPMCs and macrophages can stimulate angiogenesis through potent pro-angiogenic factors, and secretory DPMCs, macrophages and T cells have also been noted to affect adjacent non-endothelial cells or to recruit each other *via* chemotactic signals, and these potential factors jointly maintain the stability of the skin microenvironment (34, 35).Marvelous ultrastructural changes appeared during xenobiotics attack. Pericytes underwent tighter junctions with DMECs but did not generate the pathological changes of gap formation (9, 10, 36–40). We tend to identify it as a physiological change caused by altered local permeability pressure after needle-free injection, since pericytes play an important role in regulating microvascular permeability, which would permit more hemolymph exchange to proceed (41). Postcapillary microvenules and lymphatic vessels have been considered to be important channels for the entry and exit of immune cells into draining lymph nodes, as well as constituting an important component of DMUs. TEM evaluation revealed a large number of macrophages, d DCs and T cells clustered around postcapillary microvenules, concomitant hypertrophy and linkage rearrangement of lymphatic endothelial cells (29), which providing direct cytological evidence for the synergistic involvement of the components of DMUs in the immune response. We therefore concluded with caution that the large number of immune cells in the dermis and surrounding blood vessels during the immune response may be partly derived from blood-lymphatic exchange within the skin, as these cells are abundant in normal cutaneous lymphatic vessels and rare around microvessels.

DPMCs, as immune sentinel cells within the skin, have been found to degranulate and migrate following xenobiotic attack. Neutrophils were observed after needle-free injection. Recent studies have suggested that neutrophil recruitment may be related to the synthesis of MIP-2 and tumor necrosis factor (TNF) by mast cell degranulation (42–44). Similarly, histamine released by DPMCs degranulation contributes to increased endothelial permeability and promotes hemolymph exchange. Histamine also enhances the modulatory effects of macrophages and interleukin-8 (IL-8) by increasing granulocyte-macrophage colony-stimulating factor (GM-CSF) production, which facilitates an efficient immune response (45, 46). These factors, although increased immune efficiency, may trigger unnecessary inflammation. Therefore, how transdermal immunity controls the stability of mast cell membranes to mitigate possible inflammatory responses remains to be investigated thoroughly.

TC is a novel type of interstitial cell, similar in morphological characteristics with the interstitial cells of Cajal (ICCs), and was named interstitial Cajal-Like cells (ICLCs) at a time until Popescu identified it as a completely new interstitial cell (47). According to the identification criteria proposed by Popescu, we identified TCs in DMUs (48). TCs were mainly distributed in the periphery of microvessels, separating DMUs from the dermis, and we also found TCs in the periphery of dendritic cells, T cells, mast cells, and macrophages, suggesting that TCs may be involved in the regulation of skin immunity (49). Following attack by xenobiotics, TCs were observed to produce vesicles of varying sizes, which were believed to contain proteins, lipids, microRNA (miRNA), long non-coding RNA (lncRNA) and mRNA, indicating that TCs play a crucial role in signaling skin immunity and potentially control the transcriptional activity of the cells involved (50–52).

It is worth mentioning that a mysterious cell called veil cell was proposed in early studies and it was thought to form part of the DMU (53). Veil cells were described as separating the DMU from the dermis, 3D computer reconstruction showed their cell bodies with multiple wing-like projections, and were factor XIIIa^+^ (54). TEM evaluation revealed veil cells with vesicles and candy-like protrusions, and showed non-specific morphological changes in response to microenvironmental alterations (55, 56). Since TCs had not been discovered at that time and the characteristics of veil cells were similar to TCs, we cautiously inferred that veil cells might be the same type of cells as TCs, a speculation that needs further proof by immunological means.

In a nutshell, our results firstly identified DMUs consisting of DSVPs and capillary lymphatics in domestic pig skin, which occur in the dermis stratum papillare and confirmed as reservoirs of immune cells *via* IHC. TEM evaluation was used to understand the ultrastructure of DMUs in the dermis stratum papillare, which underwent ultrastructural changes and migration of immune cells after attack with xenobiotics. These changes supported the involvement of lymphatic vessels and microvessels as a whole unit in the cutaneous immune response. The role of lymphatic vessels during jet needle-free injection may be substantial because of their uncharacteristic endothelial ultrastructural changes. Evidence to date is lacking to confirm the role arising from endothelial changes in lymphatic vessels, which is potentially fertile ground for cutaneous immune studies. TCs was found to produces heterogeneous junctions with DPMCs, T cells, d DCs and DMECs in DMUs and separates microvessels from dermal collagen fibers. The ultramorphology of TCs resembles that of veil cells around microvessels in the early literature, unfortunately, we did not further demonstrate whether they are the same type of cells. This study provides morphological evidence for DMUs as reservoirs of immune cells and channels of passive immunity in the skin, and morphological evidence for the possible role of TCs in regulating the function of DMUs is also revealed.

## Data Availability Statement

The original contributions presented in the study are included in the article/supplementary material, further inquiries can be directed to the corresponding authors.

## Ethics Statement

The animal study was reviewed and approved by the Animal Ethics Committee of Nanjing Agricultural University.

## Author Contributions

XM, ZZ, and PY: conceptualization, data curation, and writing—review and editing. BD, QC, PY, and YL: formal analysis. XM, ZZ, NA, QM, QW, and PY: methodology. XM and ZZ: writing—original draft. All authors have read and agreed to the published version of the manuscript.

## Funding

The authors disclosed receipt of the following financial support for the research, authorship, and/or publication of this article: this work was supported by the Key Research and Development Program of Jiangsu Province (Grant Number: BE2020336), the Nanjing Agricultural University Student Research Training Project Fund (Grant Number: 202110307114Y), and the Priority Academic Program Development of Jiangsu Higher Education Institutions, China.

## Conflict of Interest

The authors declare that the research was conducted in the absence of any commercial or financial relationships that could be construed as a potential conflict of interest.

## Publisher's Note

All claims expressed in this article are solely those of the authors and do not necessarily represent those of their affiliated organizations, or those of the publisher, the editors and the reviewers. Any product that may be evaluated in this article, or claim that may be made by its manufacturer, is not guaranteed or endorsed by the publisher.
